# Arrhythmias in Systemic Sclerosis: A Call for Interdisciplinarity Teams

**DOI:** 10.3390/life15101608

**Published:** 2025-10-16

**Authors:** Diana Elena Costan, Veronica Ungurean, Monica Claudia Dobos, Anca Ouatu, Paula Cristina Morariu, Alexandru Florinel Oancea, Maria Mihaela Godun, Diana-Elena Floria, Dragos Traian Marcu, Genoveva Livia Baroi, Silviu Marcel Stanciu, Anton Knieling, Daniela Maria Tanase, Codrina Ancuta, Mariana Floria

**Affiliations:** 1 Department of Medical Sciences II, Grigore T. Popa University of Medicine and Pharmacy, 700115 Iasi, Romania; costan.diana0198@yahoo.com (D.E.C.); ungurean.veronica@yahoo.com (V.U.); monicabalac329@yahoo.com (M.C.D.); 2 Department of Rheumatology, Clinic Rehabilitation Hospital, 700661 Iasi, Romania; 3 Department of Medical Sciences I, Grigore T. Popa University of Medicine and Pharmacy, 700115 Iasi, Romania; anca.ouatu@umfiasi.ro (A.O.); paula.cristina-morariu@umfiasi.ro (P.C.M.); alexandru.oancea@umfiasi.ro (A.F.O.); godun.maria-mihaela@d.umfiasi.ro (M.M.G.); diana-elena.iov@d.umfiasi.ro (D.-E.F.); marius-traian-dragos.dm-marcu@umfiasi.ro (D.T.M.); floria.mariana@umfiasi.ro (M.F.); 4 Department of Internal Medicine, Sf. Spiridon County Clinical Emergency Hospital, 700111 Iasi, Romania; 5 Department of Surgery, Faculty of Medicine, Grigore T. Popa University of Medicine and Pharmacy, 700115 Iasi, Romania; livia.baroi@umfiasi.ro; 6 Department of Internal Medicine and Gastroenterology, Carol Davila University of Medicine and Pharmacy, Central Military Emergency University Hospital, 010825 Bucharest, Romania; silviu.stanciu@umfcd.ro; 7 Discipline of Forensic Medicine, Faculty of Medicine, Grigore T. Popa University of Medicine and Pharmacy, 700115 Iasi, Romania; anton.knieling@umfiasi.ro

**Keywords:** systemic sclerosis, scleroderma, arrhythmias, atrial fibrillation, ventricular fibrillation, atrial ectopic beats, ventricular ectopic beats

## Abstract

Background: Systemic sclerosis (SSc) is a rare autoimmune disease characterized by progressive fibrosis, systemic inflammation and vascular dysfunction, with manifestations that can affect multiple organs, including the heart. Cardiac involvement in SSc is often underdiagnosed, although it can have serious consequences on the prognosis, especially the occurrence of arrhythmias. These rhythm disturbances can result from direct damage to the myocardium, the conduction system, or the coronary microcirculation. Equally, the medication used can have iatrogenic consequences manifested by severe arrhythmias. Methodology: The aim of this study was to provide a synthesis of incidence, pathogenic mechanisms, diagnostic methods, and therapeutic strategies of arrhythmias associated with SSc. The potential effects of immunomodulatory therapies, such as conventional immunosuppressants and biological therapies, on cardiac electrical function were also analyzed. This narrative review could present the state of the art on arrhythmias associated with SSc, which could serve as a practical guide. In clinical practice, it is necessary to establish a team that includes cardiologists and rheumatologists as well as other specialists to contribute to a correct diagnosis followed by an optimal therapy in patients with SSc. Results: Current data suggest that diffuse myocardial fibrosis, silent ischemia, and inflammatory infiltration may alter the propagation of the electrical impulse in the heart, favoring the occurrence of arrhythmias. Atrioventricular blocks, ventricular tachyarrhythmias, and atrial fibrillation are the most commonly reported rhythm abnormalities in SSc. Also, some therapies used in the treatment of the disease may influence the arrhythmic risk. Conclusions: Cardiac arrhythmias in SSc can have a significant impact on the prognosis of patients, which is why a multidisciplinary approach is essential. Collaboration between rheumatologists, cardiologists, and electrophysiologists is crucial for the early identification and appropriate management of arrhythmic risk in this patient group.

## 1. Introduction

Systemic sclerosis (SSc) or scleroderma is a complex autoimmune connective tissue disease characterized by aberrant, extensive, and frequently irreversible fibrosis of the skin and internal organs, vascular abnormalities, and immune system dysfunctions [1]. In fact, despite the general irreversibility of fibrosis and the dated nature of the data, the 1984 study by Carol Black et al. provided compelling evidence that cutaneous manifestations in systemic sclerosis are often reversible, with spontaneous regression of skin thickening being not uncommon [2]. The disease most frequently affects the lungs, heart, and gastrointestinal system, leading to systemic involvement [1]. Nowadays, because SSc is a complex disease which touches multiple organs, a multidisciplinary team is mandatory. This narrative review aims to synthesize the main data from the literature on everything a clinician who comes into contact with such a complex pathology needs to know. Starting from epidemiology and passing through pathophysiology and diagnostic methods, this manuscript also brings to the fore therapeutic methods and the need for collaboration between multiple specializations for a favorable outcome on the prognosis of the patient with SSc. These patients are often hospitalized in internal medicine departments. Collaboration with the rheumatologist, dermatologist, cardiologist, gastroenterologist, family doctor and any other specialization is essential.

Autoimmune rheumatologic conditions such as rheumatoid arthritis, systemic lupus erythematosus, and SSc have been studied and found to be associated with an increased risk of developing cardiac, supraventricular and ventricular arrhythmias. Clinically, these rhythm disorders are often observed and contribute to a higher cardiovascular risk and also to patient mortality [3].

## 2. Materials and Methods

### 2.1. Study Design

This paper was designed as a narrative review aimed integrating and critically summarizing the newest insights into the epidemiology, pathophysiology, diagnosis, and management of arrhythmias identified in patients with SSc, as well as to analyze the cardiovascular effects of immunomodulatory and biological therapies used in the treatment of this complex autoimmune disease.

### 2.2. Search Strategy

Literature sources were selected following a structured search in the PubMed and Google Scholar databases, using combinations of Medical Subject Headings (MeSH) and free-text relevant terms such as (“systemic sclerosis” OR “scleroderma”), AND “cardiac involvement “ OR “arrhythmia” OR “atrial fibrillation” OR “conduction abnormalities” OR “ventricular arrhythmias” OR “myocardial fibrosis” OR “autonomic dysfunction” OR “microvascular ischemia”, OR “endothelial dysfunction” OR “immunosuppressive therapy” OR “biologic agents”. To ensure the timeliness and clinical relevance of the information presented, articles published in the last ten years (2015–2025) were prioritized.

### 2.3. Study Selection and Data Extraction

Following the application of exclusion criteria-namely, studies reporting arrhythmias secondary to electrolyte imbalances, endocrine disorders, or genetic conditions; studies that failed to address the primary outcomes of interest; those not aligned with the specific scope of the manuscript; and duplicate publications—98 articles were initially retained. Subsequently, an additional 13 case reports and 10 studies that focused primarily on inflammation, without demonstrating a direct causal relationship with arrhythmias, were also excluded. Ultimately, a total of 75 articles were selected for inclusion in this narrative review. These comprised 26 cohort studies investigating arrhythmias in SSc, 44 studies examining cardiovascular disease in the context of SSc, and 5 review articles (4 systematic reviews and 1 narrative review), forming a solid foundation for evaluating the interplay between inflammation, pharmacological intervention, and arrhythmic risk in this patient population.

## 3. Epidemiology of Systemic Sclerosis

Accurately defining the epidemiological burden of SSc is crucial for enhancing disease awareness, guiding healthcare planning, and optimizing clinical management. Over recent decades, a growing number of population-based studies have sought to quantify the incidence and prevalence of SSc across diverse geographic regions revealing notable differences related to ethnicity, environmental factors, and diagnostic methodology. A comprehensive meta-analysis published in 2021 screened 6983 unique records, and included 61 prevalence studies and 39 incidence studies estimating a pooled global prevalence of 17.6 cases per 100,000 individuals, and an overall incidence rate of 1.4 per 100,000 person-years. Importantly, patients with SSc exhibit a higher prevalence of arrhythmic complications compared to those with other autoimmune diseases, underscoring the importance of routine cardiovascular monitoring in this population [4].

The most common arrhythmias described are atrial fibrillation, atrial flutter, paroxysmal supraventricular tachycardia, in a proportion of 20–30% while ventricular arrhythmias are present in up to 67% of patients [5]. Premature ventricular contractions are also observed, the most frequent being the monomorphic forms. They can appear isolated or less often as bigeminy, trigeminy or couplets [3]. Also, sudden cardiac death, a sensitive topic, has been reported in around 21% of patients with SSc, underlining the need for early diagnosis and management of cardiac complications in this group of patients [6].

## 4. Pathophysiology of Arrhythmias in Systemic Sclerosis

In SSc, persistent inflammation plays a central role, leading to the development and maintenance of myocardial fibrosis, which is a key factor in arrhythmogenesis. The fibrotic remodeling present in the myocardium can cause diffuse damage, impairing the integrity of the myocardial conduction function and ultimately resulting in arrhythmias [7]. When fibrosis involves components of the conduction system as the sinoatrial node and the His-Purkinje network, bradyarrhythmia and conduction disturbances may occur [8]. Another mechanism that sustains arrhythmic risk in SSc is autonomic nervous system dysfunction. In this group of patients, marked rhythm instability has been observed, due to a sympathetic-parasympathetic imbalance facilitates the occurrence of arrhythmia [5]. Clinically, cardiac magnetic resonance imaging (CMR) is a valuable method for detecting myocardial damage in patients with SSc. It is considered an important tool because it provides high-resolution assessment of myocardial fibrosis and identification of arrhythmogenic areas, thus supporting early therapeutic management in these patients [7].

**Figure 1:**
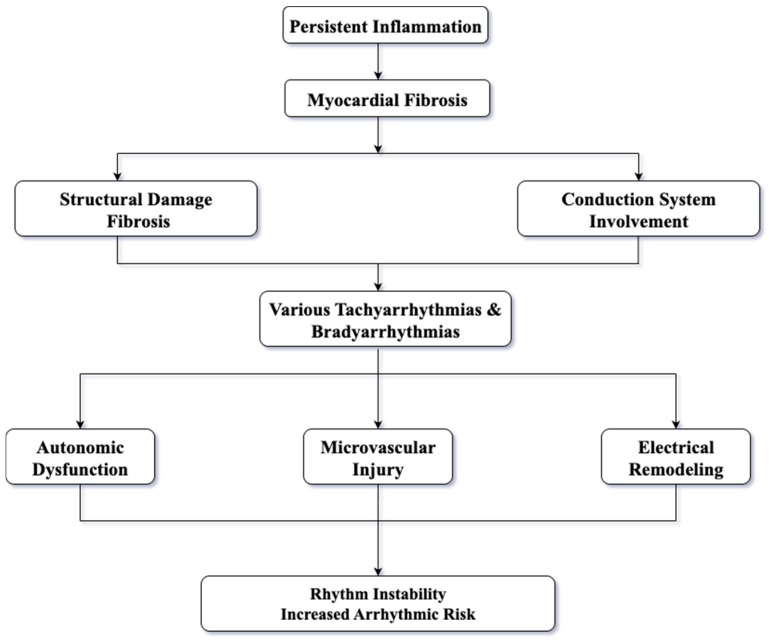
The mechanisms involved in arrhythmogenesis.

Histopathological consistently shows replacement of myocardial tissue by fibrosis; these findings have been correlated with the clinical occurrence of rhythm disturbances [9]. At the molecular level, processes such as collagen synthesis, immune activation, structural remodeling, and electrical vulnerability are driven by dysregulated pathways. These mechanisms represent potential therapeutic targets to reduce the arrhythmic burden in this high-risk population [10]. Arrhythmogenicity is determined mainly by the formation of a structural substrate due to the atrial and/or ventricular fibrosis- referred to as atrial and ventricular structural remodeling. Electrical remodeling of the heart also plays a key role. The main mechanisms involved are complex, with evidence suggesting the implication of autonomic nervous system (ANS), renin–angiotensin–aldosterone system (RAAS), endothelial dysfunction, epicardial tissue adiposity or inflammation. Figure 1 illustrates the main mechanisms involved in arrhythmogenesis both in general and, in particular, in SSc.

### 4.1. Atrial and Ventricular Remodeling and Arrythmogenicity

SSc is frequently associated with intrinsic cardiac involvement, which is related to both atrial and ventricular remodeling. The origin of these cardiac changes is multifactorial, mainly resulting from progressive myocardial fibrosis, small-vessel vasculopathy, and chronic inflammatory processes. Fibrous tissue replacement together with microvascular ischemia, leads to disruption of normal myocardial architecture and function. The consequences are diastolic dysfunction, impaired contractility, and conduction abnormalities, which may occur even in the absence of cardiovascular symptoms. Atrial remodeling, particularly, predisposes patients to arrhythmias, while ventricular changes may worsen heart failure and reduce cardiac reserve, underlining the clinical importance of early cardiac evaluation in SSc [11].

In a study involving 74 participants, including 34 patients diagnosed with SSc and 34 healthy control subjects, two-dimensional speckle-tracking echocardiography, an advanced cardiac imaging technique, was used. Compared with conventional echocardiography, two-dimensional speckle-tracking echocardiography can detect subtle ventricular dysfunction that might otherwise go unnoticed [12]. The results showed significant atrial involvement in the SSc group, with evidence of impaired interatrial conduction due to pathophysiological mechanisms specific to the disease. In addition, tissue Doppler imaging revealed higher systolic pulmonary artery pressure and increased mitral A-wave velocities, along with a lower left ventricular filling flow E/A ratio–these findings suggest early diastolic dysfunction in this patient group [13].

Impaired right atrial function may also develop as a consequence of increased systolic pulmonary artery pressure, often accompanied by right ventricular hypertrophy. In the context of pulmonary arterial hypertension, right atrial augmentation function becomes especially important, as it plays a key role in maintaining adequate right ventricular filling. Both systolic and diastolic atrial dysfunction can increase mean atrial pressures, potentially leading to pulmonary or systemic venous hypertension [12,14].

### 4.2. Autonomic Nervous System and Arrhythmogenicity

The sympathetic-parasympathetic imbalance was observed in SSc patients. Increased sympathetic drive combined with reduced parasympathetic activity creates a pro-arrhythmic state, favoring the development of various conduction and rhythm disturbance. Dysregulation of the ANS occurs frequently and often early in patients with SSc, playing a trigger role in the development of cardiac arrhythmias, through a sympathetic-parasympathetic imbalance, increasing the risk of sudden cardiac death. From an electrophysiological standpoint, inflammation and the subsequent development of myocardial fibrosis represent the cornerstone of arrhythmogenesis in SSc. Nonetheless, without concomitant ANS dysfunction encompassing both sympathetic and parasympathetic branches, the arrhythmias would be unlikely to manifest. While myocardial fibrosis provides the anatomical substrate for reentry mechanisms, ANS imbalance serves as a potent arrhythmic trigger. In this context, both elements, fibrosis and autonomic dysregulation, can be implicated in the arrhythmogenic process. Multiple studies have shown that patients with SSc frequently present autonomic dysfunction, often reflected by decreased heart rate variability and abnormal heart rate turbulence. These changes indicate a reduced ability to maintain adequate cardiac regulation, which may precede clinical cardiac symptoms [15].

In a study involving 45 patients with SSc and 30 healthy controls, significant abnormalities in heart rate turbulence parameters were observed in the SSc group, suggesting early autonomic involvement. Age and the presence of pulmonary hypertension were identified as independent predictors of autonomic dysfunction [15]. Another study found a relationship between autonomic dysfunction and microvascular damage in SSc. Nailfold video capillaroscopy demonstrated that patients with severe microvascular abnormalities are more likely to present signs of cardiac autonomic neuropathy [16].

One study also found a significant positive association between diastolic dysfunction–affecting either the left or right ventricle–and total skin score and the presence of anti-Scl-70 antibodies. In contrast, there was an inverse relationship between diastolic dysfunction and several parameters of heart rate variability, except for the low-frequency/high-frequency ratio (known as LF/HF), which was positively correlated. No significant correlation was found between diastolic dysfunction and the presence of anticentromere antibodies. In addition, cardiac rhythm disturbances were documented in a significant proportion of patients with SSc. In a 2010 study involving 30 patients with SSc, 33% of patients presented frequent premature ventricular contractions and 7% experienced episodes of supraventricular tachycardia, while in the control group arrhythmias were rarely detected. Moreover, the total premature ventricular contractions burden was considerably higher in patients with SSc compared to healthy controls [17].

Beyond its direct effects on the myocardium, autonomic dysregulation in SSc impacts multiple organ systems. Among the most frequently affected are gastrointestinal mobility and thermoregulation. In a 2021 cross-sectional study involving 26 patients diagnosed with SSc, a high proportion presented with Raynaud’s phenomenon (80%) and esophageal dysmotility (43%), highlighting the significant systemic impact of ANS dysfunction [16].

These findings support the hypothesis that the appearance or exacerbation of Raynaud’s phenomena may represent one of the earliest clinical manifestations of autonomic dysfunction, particularly from a microvascular perspective. Similarly, esophageal motility disorders have been associated with cardiac autonomic neuropathy, suggesting that autonomic involvement may occur early in the disease course, preceding myocardial fibrosis or other internal organ complications [17].

### 4.3. Renin–Angiotensin–Aldosterone System and Arrhythmogenicity

The renin–angiotensin-aldosterone system plays a central role in maintaining blood pressure and fluid–electrolyte balance. The kidneys represent the main source of prorenin, which is converted to renin and secreted by the juxtaglomerular cells in response to reduced renal perfusion, low sodium delivery to the *macula densa*, β_1_-adrenergic stimulation. Then, renin cleaves Angiotensinogen, produced by the liver, into Angiotensin I (Ang I) which is rapidly converted into Angiotensin II by angiotensin-converting enzyme. The ACE is predominantly located in the pulmonary and vascular endothelium [18,19]. Angiotensin II is a potent vasoconstrictive peptide and exerts its effects mainly via two receptor subtypes: angiotensin II type 1 receptor (AT_1_R), which predominates in adults and mediates vasoconstriction by increasing intracellular calcium in vascular smooth muscle cells and angiotensin II type 2 receptor (AT_2_R) which mediates vasodilation and tissue repair through nitric oxide release [20,21]. Aldosterone, the terminal effector of the RAAS cascade, promotes sodium retention and contributes to tissue fibrosis by stimulating the synthesis of extracellular matrix components such as fibronectin and type I collagen [21].

In SSc, the pathogenic role of RAAS is amplified by functional autoantibodies against AT_1_R and endothelin receptor type A. These antibodies activate endothelial cells, increasing the production of chemokine interleukin-8 (IL-8) and vascular cell adhesion molecule-1, enhancing neutrophil migration and impairing endothelial repair. They also stimulate fibroblasts to produce type I collagen, favoring fibrosis. Experimental studies have shown that transferring these autoantibodies to mice triggers lung inflammation, elevated chemokine levels and histological changes associated with systemic vascular injury [22,23]. The clinical evidences shows that RAAS dysregulation is relevant for cardiac manifestations in SSc. In a cohort of 110 patients, approximately 61% exhibited conduction or rhythm disturbances- from premature ventricular contractions to atrioventricular blocks and atrial arrhythmias which were frequently associated with pulmonary arterial hypertension and myocardial fibrosis. These findings support hypothesis that RAAS activation contributes to adverse myocardial remodeling, creating an arrhythmogenic substrate in SSc patients [24].

### 4.4. Endothelial Dysfunction and Arrhythmogenicity

Endothelial dysfunction is key in developing large-vessel atherosclerosis. Even early on in SSc, the small blood vessels’ lining shows damage or activation due to factors like immune attacks, infections causing cell death, antibodies targeting the endothelium, and injury from blood flow changes [25]. Higher endothelin levels—a strong vasoconstrictor made by these cells—make this problem worse. This contributes to blood vessel disease seen in SSc and speeds up atherosclerosis. As a result, patients with SSc have a higher chance of problems like peripheral artery disease, strokes, carotid artery issues, and heart disease [26].

### 4.5. Epicardial Tissue Adiposity, Inflammation and Arrhythmogenicity

Epicardial adipose tissue (EAT) surrounds the heart and coronary vessels, acting like a protective cushion under normal conditions. It helps reduce mechanical stress on the heart and supports blood vessel remodeling. EAT also plays a role in keeping the heart warm by producing heat [27]. Adipose tissue generally comes in two types: white and brown fat. White fat stores energy in large droplets and has few mitochondria, while brown fat has many small droplets and lots of mitochondria to help produce heat. Although EAT is mainly white fat, it shows some brown and beige fat-like features. It expresses thermogenic genes and releases various inflammatory and anti-inflammatory mediators, including TNF-α, interleukins, C-reactive protein, and plasminogen activator inhibitor-1 [28].

Clinical studies have found that in SSc, having a higher amount of EAT-over 67 g-is linked to worse left ventricular diastolic function and higher overall mortality over an 8-year period. These findings suggest that excess EAT contributes to both structural and functional changes in the myocardium in SSc [29]. Atrial fibrillation is the most common heart rhythm disorder seen in clinical practice and is a major cause of ischemic stroke, heart failure, and cardiovascular deaths. It develops because of complex electrophysiological and structural changes within the atria, notably myocardial fibrosis involving multiple mechanisms. Factors that promote atrial fibrillation include adipocyte infiltration, paracrine signaling with pro-fibrotic and pro-inflammatory effects, oxidative stress, and other interconnected pathways [30].

### 4.6. Potential Cardiovascular Risk Factors and Arrhythmogenicity

In SSc the risk of developing arrhythmias is driven by a series of mechanisms involving structural, functional, and electrical abnormalities, many of which are triggered by ongoing inflammation and tissue damage. Several of these risk factors have already been discussed in the previous sections. Table 1 brings them together for a clear overview as a reminder of their combined role leading to arrhythmogenesis. The first feature, autonomic dysfunction, caused by an imbalance between the sympathetic and parasympathetic nervous systems, can lead to abnormal rhythms and even sudden cardiac death [17]. Myocardial fibrosis, driven by chronic inflammation and collagen buildup, makes the heart muscle stiffer, which can lead to restrictive cardiomyopathy and heart failure. Also, some of medications used to treat SSc (corticosteroids, prostacyclin analogues, biologics, etc.) can have cardiotoxic or arrhythmogenic effects, which highlights the need to weigh their therapeutic benefits against possible cardiovascular risks [31]. The damage of both micro and macro blood vessels-often linked to coronary artery involvement and endothelial dysfunction-can significantly reduce blood flow to the heart [32,33].

At the same time, chronic inflammation, kidney and digestive system involvement, along with side effects from medications, can lead to electrolytes imbalances-making the heart more vulnerable to rhythm disturbances, including torsade de pointes, ventricular tachycardia (VT), or even cardiac arrest. Sadly, these complications are not rare-around 26% of patients with SSc lose their lives due to heart-related causes, most often because of heart failure or severe arrhythmias [34].

### 4.7. Effects of Drug Used in the Treatment of Systemic Sclerosis and Cardiac Involvement

Managing SSc is a complex process that requires a complex and personalized approach. Treatment focuses on several key areas: correcting the immune dysregulation, reducing or preventing fibrosis to help preserve tissue and organ function, and improving skin symptoms through both systemic and local treatments, as well as targeting blood vessel abnormalities. Alongside the main aims, it’s also essential to prevent and manage potential complications involving the internal organs—particularly the lungs, heart, gastrointestinal tract, and kidneys—while controlling manifestations and improving quality of life. Because SSc may have multisystemic involvement is often required a multidisciplinary team and an individualized treatment plan.

**Figure 2:**
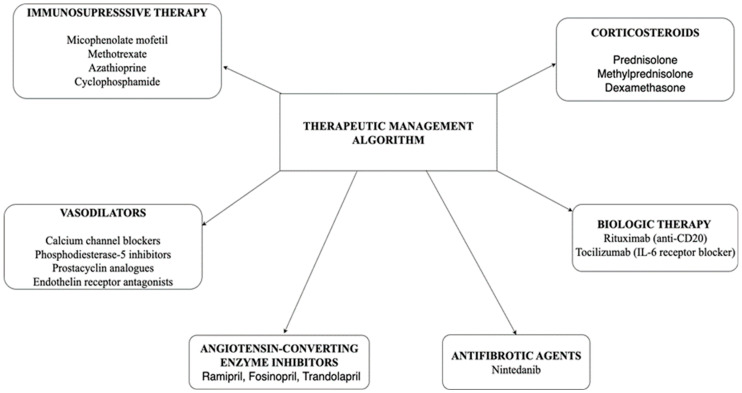
Therapeutic management algorithm in systemic sclerosis (SSc). IL: interleukin.

In Figure 2 the therapeutic management algorithm in SSc is described, illustrating therapeutic classes such as immunosuppressive medication, vasodilators, antifibrotic agents and targeted biologic therapies. The specific combination and choice of drugs depend on the type of SSc, the severity and pattern of organ involvement. These treatment options will be discussed in more detail in the following sections.

#### 4.7.1. Immunosuppresssive Medication

**Mycophenolate mofetil (MMF)** is a recognized immunosuppressant used in SSc. Its mechanism of action involves inhibiting lymphocyte proliferation by blocking inosine monophosphate dehydrogenase, an enzyme essential for DNA synthesis in T and B cells. It is currently considered the first-line therapeutic option, particularly in SSc-associated interstitial lung disease (SSc-ILD), owing to its demonstrated efficacy and favorable safety profile compared to alternative immunosuppressive agents. Additionally, MMF is appreciated for its potential to stabilize or even improve cutaneous involvement [35].

Its clinical utility in SSc-ILD has been supported by multiple studies, including a real-world study in 2017 [36]. The study evaluated 46 patients receiving a reduced dose of 3 g/day and found that, over a 24-month treatment period, pulmonary function parameters remained relatively stable. Moreover, systolic pulmonary artery pressure (RVSP) showed no significant change, and the therapy was generally well tolerated, with minimal adverse effects. Nonetheless, potential arrhythmogenic effects of MMF have also been reported. In a case report involving a 55-year-old patient undergoing MMF therapy, Holter ECG monitoring revealed the presence of both ventricular and atrial ectopic beats, an episode of supraventricular tachycardia, as well as bundle branch blocks [37].

**Methotrexate (MTX)** is an antifolate antimetabolite whose mechanism of action involves inhibition of dihydrofolate reductase, resulting in impaired DNA synthesis, as well as suppression of purine and pyrimidine production, thereby exerting anti-inflammatory effects. In SSc, methotrexate is primarily used in the diffuse cutaneous form, although its impact on skin thickening is generally modest [38]. As methotrexate is considered an alternative, rather than first-line, treatment in SSc, current evidence regarding its cardiovascular safety remains limited. Nevertheless, it appears to have a favorable safety profile in many patients. However, in isolated reports—such as the case described by Sareena Shah et al. (2022) [39]—methotrexate has been associated with cardiotoxic effects. Documented adverse events have included ventricular arrhythmias, right bundle branch block, and other idiosyncratic rhythm disturbances [39]. In conclusion, although such cases are rare, they underscore the importance of careful cardiovascular monitoring during Methotrexate therapy, as adverse effects—though infrequent—may include significant arrhythmias or, in severe cases, heart failure.

**Azathioprine (AZA)** is a pro-drug of 6-mercaptopurine (6-MP) which is converter into thioinosinic acid, leading to inhibit purine synthesis, reducing DNA and RNA production-the final result being the T and B cell suppression. AZA is considered a therapeutic alternative for patients with SSc who do not respond to, or cannot tolerate, other immunosuppressive treatments. A literature review published in 2025, which included 10 studies, analyzed the effects of Azathioprine on both cutaneous and pulmonary parameters. The results were generally positive, particularly in patients with SSc-ILD and also in those who had previously received cyclophosphamide as part of their initial treatment option [40]. To date, the effects of azathioprine on cardiac rhythm disturbances in patients with SSc have not been clearly defined, as no dedicated studies have addressed this aspect.

**Cyclophosphamide (CYC)** is an alkylating immunosuppressant that works by cross-linking DNA, leading to the death of rapidly dividing immune cells. In SSc, this mechanism helps reduce inflammation and slow the progression of fibrosis. For many years, cyclophosphamide has been a key treatment in SSc and remains the first-line therapy for SSc-ILD, according to the European League Against Rheumatism (EULAR) recommendations. However, due to its significant toxicity compared to MMF—and similar efficacy in treating both skin and lung involvement—MMF is often preferred as a first-line treatment based on clinical experience and expert opinion [41]. Although no studies have clearly demonstrated a direct link between cyclophosphamide and arrhythmias specifically in patients with SSc, some of its known general pro-arrhythmic effects include: atrial fibrillation, supraventricular tachycardia, premature ventricular contractions, VT, and in high-dose can lead to high-grade atrioventricular block, also carrying cardiotoxic effects [42].

Among current treatment options, mycophenolate mofetil is frequently preferred, primarily due to its more favorable safety profile compared to the known cardiotoxic risks associated with Cyclophosphamide. Evidence from prospective cohort studies supports its use, demonstrating improvements in cardiac function and overall clinical outcomes. MMF has shown efficacy both as a first-line therapy in systemic rheumatic conditions like SSc, and as a second-line agent in patients with isolated, virus-negative or autoimmune lymphocytic myocarditis—particularly in those who do not tolerate or respond to Azathioprine—regardless of the concurrent glucocorticoid dosage [31]. In SSc, cardiac involvement often leads to arrhythmias, significantly impacting morbidity and mortality. Anti-inflammatory treatments, especially corticosteroids, are frequently used to manage systemic inflammation in SSc. Immunosuppressive agents (e.g., methotrexate and cyclophosphamide) are crucial for disease control but have variable effects on cardiac conduction and arrhythmia risk. Methotrexate may rarely induce ventricular arrhythmias. Overall, while inflammation control is essential, these therapies require careful cardiac monitoring due to their potential pro-arrhythmic effects. Further research is needed to clarify their impact on arrhythmia risk in SSc patients [31].

The main immunosuppressive agents used in SSc, together with their cardiovascular implications, are summarized in Table 2.

#### 4.7.2. Corticosteroid Therapy

Corticosteroids such as prednisolone, methylprednisolone, and dexamethasone, continue to play a role in managing SSc and other rheumatic conditions, thanks to their well-established anti-inflammatory and immunosuppressive properties. They exert their effects by modulating the activity of key immune cells, including macrophages, T cells, and B cells, and by influencing fibroblasts and endothelial cells, both central to the development and progression of the disease. Given their well-documented anti-inflammatory capacity and regulate immune responses, corticosteroids can help control the complex processes that drive persistent inflammation, vascular dysfunction, and fibrosis in SSc. While their use requires careful balancing of benefits and risks, they remain a valuable option in selected clinical settings [43].

Corticosteroid therapy can have notable effects on cardiovascular function. A 2015 review documented cases of bradycardia occurring after high-dose pulse therapy with prednisolone or its equivalent, specifically at doses exceeding 250 mg per day [44]. Additionally, a 2013 study highlighted that ventricular arrhythmias—including premature ventricular complexes, no sustained, and sustained VT—as well as sudden cardiac death, are frequently associated with myocardial fibrosis in patients with SSc. The widespread use of corticosteroids may exacerbate these arrhythmias through metabolic alterations and conduction disturbances such as hypokalemia and hypocalcemia. Moreover, corticosteroid treatment has been linked to an increased risk of atrial arrhythmias, including atrial fibrillation and atrial flutter [8]. A recent review published in *Rheumatology Quarterly* examined the risk of SSc renal crisis, which is the most feared complication associated with corticosteroid use in SSc. The study demonstrated that administering corticosteroids at doses greater than 15 mg/day for at least six months increases the risk of SSc renal crisis to 36% in patients with diffuse cutaneous SSc (dcSSc) [45]. The evidence regarding corticosteroid therapy used and the effects on SSc and cardiovascular is presented in Table 3.

#### 4.7.3. Vasodilators (Calcium Channel Blockers, Phosphodiesterase-5 Inhibitors, Prostacyclin Analogues, and Endothelin Receptor Antagonists)

Vasodilators such as calcium channel blockers (e.g., Nifedipine and Amlodipine), phosphodiesterase-5 inhibitors (e.g., Sildenafil and Tadanafil), prostacyclin analogues (e.g., Iloprost) and endothelin receptor antagonists (e.g., Bosentan and Ambrisentan) are used in SSc to treat vascular complications like Raynaud’s phenomenon, digital ulcers and pulmonary hypertension. Calcium channel blockers are considered first-line treatment for Raynaud’s phenomenon and digital ulcers, while phosphodiesterase-5 inhibitors are considered second-line treatment for digital ulcers, but are also indicated in the treatment of pulmonary hypertension. They are contraindicated in unstable angina and recent history of myocardial infarction [46,47].

A 2006 study involving 21 patients with SSc, including 17 women, showed that Iloprost exerts cardiovascular effects, such as prolongation of the ventricular refractory period and QTc interval, as well as myocardial ischemia through a ‘coronary steal’ phenomenon [47]. Thus, it is contraindicated in patients with severe coronary artery disease, a history of thrombotic events, congestive heart failure, or patients known to have severe arrhythmias, all of which may be aggravated by Iloprost [48].

Bosentan has shown encouraging results in patients with SSc, particularly in the management of digital ulcers. A recent retrospective analysis from 2025 involving 727 patients without pulmonary hypertension found that those receiving Bosentan for digital ulcers had a lower risk of developing pulmonary hypertension over a two-year period [49,50]. Earlier, a 2011 study with 188 patients reported a 30% reduction in the number of new ulcers after 24 weeks of treatment, especially in those with multiple lesions [51]. Another study involving 77 patients revealed that individuals with elevated endothelin-1, asymmetric dimethylarginine, and VEGF levels were more likely to experience severe microvascular complications, even while on treatment with Bosentan [52].

Dihydropyridine calcium channel blockers, in particular, have minimal negative inotropic effects and are generally well tolerated, with reflex tachycardia and lower limb edema being the most common side effect [53].

An exploratory analysis published in 2024, which included 1048 patients with SSc from 42 hospitals across France, reported a decrease in the incidence of diastolic dysfunction and an improvement in left ventricular ejection fraction below 50% after three years of treatment with vasodilators, specifically sildenafil. These findings suggest a potential protective effect of sildenafil on cardiac function in patients with SSc [46].

The most reported cardiovascular adverse effects associated with these agents include tachycardia, arterial hypotension, palpitations, and episodes of syncope [48]. Regarding arrhythmogenic risk, some sources suggest that it is minimal; however, recent studies directly addressing this issue are lacking. To date, no conclusive evidence confirms the arrhythmogenic potential of the drug, and the topic remains insufficiently explored in the literature. Table 4 highlights vasodilator therapies with action on SSc and potential benefits/risks on cardiovascular system.

#### 4.7.4. Angiotensin-Converting Enzyme Inhibitors

Angiotensin-converting enzyme inhibitors (e.g., Enalapril, Lisinopril, Ramipril, Fosinorpil, Trandolapril, etc.) represent the cornerstone of antihypertensive therapy in SSc renal crisis. Treatment should begin as soon as the diagnosis is confirmed, or the dosage increased if the patient is already on an ACE inhibitor [53].

A 2022 meta-analysis including nine studies found that prior use of ACE inhibitors in patients with SSc was associated with an increased risk of developing SSc renal crisis, with a reported risk ratio of 2.05. Furthermore, prognosis was poorer in these patients, with a risk ratio of 1.46, compared to those who began ACE inhibitor therapy at the time of SSc renal crisis diagnosis [54]. Resistance to these drugs is not uncommon in patients with SSc, making gradual dose escalation necessary, often up to the maximum tolerated dose [53]. Notably, ACE inhibitors have dramatically improved survival rates in SSc renal crisis, reducing one-year mortality from 85% to 24%, and five-year mortality from 90% to 35%, underscoring their critical role in management [48]. The role of ACE inhibitors in SSc, but also the cardiac effects is presented in Table 5.

#### 4.7.5. Antifibrotic Agents (Nonreading)

Nintedanib is an antifibrotic agent approved for the treatment of SSc-associated interstitial lung disease (SSc-ILD), known to slow the progression of lung function decline. Its efficacy has been supported by several studies, including a 2022 trial comparing two patient groups: 197 in the SENSCIS-ON (continuing treatment) group and 247 in the SENSCIS (newly initiated) group. Over 52 weeks, patients in the SENSCIS-ON group showed a smaller average decline in forced capacity vital (−58.3 ± 15 mL) compared to the SENSCIS group (−44 ± 16.2 mL), highlighting both the effectiveness and sustained benefits of ongoing Nintedanib therapy [55]. Derrick Herman et al. (2024) [56] realized a meta-analysis examining the efficacy of Nintedanib as monotherapy compared to its use in combination with mycophenolate in patients with SSc-related ILD. The combination therapy group showed a greater reduction in forced capacity vital decline—79.1 mL less compared to 46.4 mL in the monotherapy group, indicating enhanced treatment efficacy. However, this benefit came with a higher incidence of gastrointestinal side effects in patients receiving both agents [56].

Regarding the cardiac effects, including the potential for arrhythmias, data from the SCINSCIS trial from 2020 have also been considered. The study evaluated Nintedanib versus placebo in SSc-ILD and cardiovascular adverse events were rare and comparable between groups. Notably, no arrhythmias or cardiac failure episodes were reported among those receiving Nintedanib, even among patients with pulmonary hypertension at baseline [57]. A comprehensive safety analysis from the SENSCIS trial, which monitored adverse events over approximately 100 weeks, reported no cases of myocardial infarction among patients receiving Nintedanib, compared to four cases in the placebo group. Interestingly, major adverse cardiovascular events were observed less frequently in the Nintedanib group [58]. According to a pivot study published by Ninagawa et al. (2023) [59], 20 patients were ultimately included in the final analysis after applying selection criteria. Using CMR, the study observed that Nintedanib treatment was linked to a reduction in myocardial inflammation and fibrosis, reflected by a lower extracellular volume. It also showed an improvement in right ventricular function. Together, these results point toward a possible cardioprotective effect of Nintedanib, rather than any indication of cardiac toxicity [59].

The evidence of antifibrotic agents in SSc and their potential effect on cardiovascular system is summarized in Table 6.

#### 4.7.6. Biologic Therapy

Biologic therapies have become an important pivot in the management of SSc, particularly for patients who do not respond well to conventional immunosuppressive treatments. Among these, Rituximab and Tocilizumab stand out for their targeted mechanisms and growing clinical use. With more clinical experience and supporting data, both agents are gaining ground as valuable additions to treatment strategies, offering new possibilities for patients with few therapeutic alternatives.

**Rituximab**, a chimeric monoclonal antibody directed against the CD20 antigen on B lymphocytes, exerts its therapeutic effects by depleting circulating and tissue B cells, thereby attenuating autoimmune inflammation and fibrotic processes. Beyond its established efficacy in cutaneous and pulmonary manifestations of SSc, emerging evidence supports its potential role in cardiac involvement. A 2024 multicenter cohort study involving 350 patients with SSc and primary cardiac involvement evaluated the efficacity of rituximab in addition to standard immunosuppressive treatments. The results showed improvements in cardiac function and a reduction in inflammatory markers. Rituximab was generally well tolerated, without an evident increase in negative reactions relative to standard therapy alone. The authors suggest that rituximab may offer a promising adjunct treatment for patients with SSc-related myocarditis by targeting B-cell-mediated inflammation. However, larger studies are needed to confirm these findings and establish clear treatment protocols [60].

In a recent cohort study from June 2025, ten patients with refractory SSc and primary cardiac involvement, unresponsive to cyclophosphamide treatment, were initiated on rituximab in combination with mycophenolate mofetil. The results demonstrated an improvement in cardiac function and a reduction in fibrosis, suggesting a potentially beneficial cardiovascular effect. Even so, further studies are needed, particularly regarding the arrhythmogenic risk [61].

**Tocilizumab**, a blocker of the interleukin-6 receptor, has also shown potential in slowing disease progression and lowering inflammatory activity. A 2022 study aimed at evaluating the efficacy and safety of tocilizumab in patients with SSc used data from the EUSTAR registry and included 93 patients treated with tocilizumab compared to 3180 patients receiving standard therapies. Outcomes were assessed at the cutaneous and pulmonary levels after 12 ± 3 months, using the modified RODNAN skin score and forced vital capacity. The findings showed some improvement in the progression of skin thickening and pulmonary fibrosis, although the results did not reach statistical significance in terms of efficacy. Regarding cardiovascular effects, isolated cases of atrial flutter, heart failure, and sudden death were reported [62]. Also, in the context of combining tocilizumab with immunosuppressive therapy-in this case Cyclophosphamide, a case report published in July 2025 describes the clinical course of a 25-year-old female patient with a severe form of SSc. The dual therapy resulted in clear clinical improvement: cardiac symptoms stabilized, heart function improved, and laboratory markers reflecting inflammation and fibrosis declined, pointing to a positive effect on myocardial involvement. The treatment was well tolerated, and no significant cardiac adverse events, including arrhythmias, were reported [63].

In conclusion, while Rituximab and Tocilizumab are already part of current treatment approaches, several other biologic agents remain under clinical investigation. Trials involving abatacept, Belimumab, Romilkimab, and Fresolimumab are in progress to assess their safety profile, therapeutic efficacy, and effects on the immune and fibrotic mechanisms underlying SSc. The results of these studies will help define the place of these therapies in future treatment strategies, to optimize management strategies for this multifaceted disease.

In Table 2, the effects of medications in SSc are detailed, addressing both their impact on the underlying disease and on the cardiovascular system. Table 7 outlines the main biologic agents evaluated in SSc and their potential role in modulating cardiovascular involvement.

## 5. Diagnostic of Arrhythmias in Systemic Sclerosis

In clinical practice, every patient with SSc should undergo a cardiology evaluation at the time of SSc diagnosis, in order to assess the extent of cardiac involvement. Subsequently, cardiological follow-up is mandatory. An annual cardiological check-up is recommended and whenever an evolution of the disease or complaints affecting the cardiovascular system are noted. Medical history should focus on so called red flags, including features of arrhythmic syncope, e.g., an absence of vagal prodrome and a family history of premature or sudden cardiac death. Among laboratory testing, natriuretic peptides (b-type-natriuretic peptide, or N-terminal pro-b-type-natriuretic peptide) may have a role in the identification of individuals at increased risk of sudden cardiac death. The 12-lead ECG, signal-averaged ECG and ECG Holter monitoring are important tools for the diagnosis of underlying disease and for risk stratification. In selective cases, electrophysiological study and genetic testing together with cardiac imaging can improve the risk stratification in patients with malign ventricular arrhythmias. There is no specific algorithm for these patients. The cardiologist uses the current recommendations for malignant arrhythmias and sudden death to evaluate patients with SSc from the 2022 ESC Guidelines for the management of patients with ventricular arrhythmias and the prevention of sudden cardiac death to evaluate and manage these patients. For individuals with reduced left ventricular ejection fraction (≤35%) and symptomatic heart failure (NYHA class II or higher), primary prevention with an implantable cardioverter-defibrillator (ICD) is indicated. In cases where patients have experienced sustained ventricular arrhythmias or cardiac arrest, ICD implantation is recommended as part of secondary prevention [64].

### 5.1. Clinical Predictors of Cardiac Involvement

Cardiac involvement in SSc has been correlated with multiple clinical and immunological factors. These include male sex, the diffuse cutaneous subtype (dcSSc), rapid skin thickening and the presence of anti-topoisomerase I (anti-Scl70), anti-Ku, anti-Histone, anti-RNA polymerase III, and anti-U3-RNP [65,66]. Additional predictors include older age at disease onset, tendon friction rubs, digital ischemia or ulcers, interstitial lung disease, and coexisting myositis [65]. Recognition of these can help identify patients who require closer cardiologic surveillance and early diagnostic testing. In a study analyzing 216 patients with SSc, it was demonstrated that anti-Th/To antibodies were more frequently associated with pericarditis compared to anticentromere antibodies [66].

### 5.2. Symptoms

The presence of arrhythmia-related symptoms (such as palpitations, presyncope, syncope, or near-syncope) warrants further investigation through 12-lead standard ECG and short- or long-term Holter monitoring for appropriate diagnosis. However, the absence of such symptoms does not exclude the presence of rhythm or conduction disturbances. Therefore, cardiological evaluation should include, at a minimum, a 12-lead standard ECG and transthoracic echocardiography. Based on the presence or absence of symptoms, the cardiologist will determine the appropriate extent of the cardiac assessment. Ventricular tachycardia (VT) and ventricular fibrillation (VF) are life-threatening arrhythmias and major causes of sudden cardiac death. These may occur in patients with or without structural heart disease. Idiopathic premature ventricular complexes (PVCs) and VT often affect patients without underlying heart disease. Although frequently benign, high PVC burden can lead to reversible cardiomyopathy and ventricular dysfunction, warranting close monitoring and treatment. Management varies from observation in asymptomatic cases to pharmacologic therapy with antiarrhythmics like amiodarone, flecainide, or sotalol in symptomatic or high-risk patients. Catheter ablation is an effective option for refractory cases. In malignant VT and VF, antiarrhythmic drugs help control arrhythmias, but implantable cardioverter-defibrillators are essential for preventing sudden death. Beta-blockers such as metoprolol, bisoprolol, and carvedilol reduce arrhythmic risk and improve outcomes [64].

Cardiac rhythm disturbances in SSc often develop insidiously and may remain subclinical until advanced stages of disease. Early identification of arrhythmogenic risk is therefore essential for preventing sudden cardiac events and improving long-term prognosis. Diagnostic assessment should address both the structural substrate of myocardial injury and the functional manifestation of electrical instability [64].

### 5.3. Biomarkers of Myocardial Involvement

Regarding biochemical markers of cardiac involvement in the context of autoimmune diseases, such as SSc, BNP and NT-proBNP have proven to be reliable indicators of myocardial dysfunction. In patient cohorts with SSc, elevated NT-proBNP levels have been associated with more severe valvular disease and a reduced left ventricular ejection fraction compared to the healthy population. Troponins I and T, particularly high-sensitivity variants, serve as valuable adjunctive biochemical markers for assessing the extent of myocardial involvement. They have demonstrated high accuracy in identifying cardiovascular damage within the context of systemic autoimmune diseases [67].

### 5.4. Electrocardiographic Evaluation

The second step is to highlight the presence of arrhythmias and their severity. The diagnostic approach to arrhythmias in SSc has evolved considerably, comprising a range of modalities from standard laboratory evaluations to advanced imaging techniques. These tools seek to identify both structural and functional cardiac abnormalities associated with arrhythmogenic risk in this patient group.

Among the standard tools for cardiovascular assessment, ECG remains the most frequently employed and essential method for detecting arrhythmias. Common ECG findings in patients with autoimmune diseases include QT interval prolongation, bundle branch blocks, and ventricular hypertrophy [31]. The 24-h Holter ECG monitoring has become one of the most used methods in providing a more comprehensive evaluation of arrhythmic occurrence, particularly in detecting arrhythmias that may go unnoticed during a standard ECG. This method can identify supraventricular arrhythmias, including atrial fibrillation, which often necessitates prompt intervention, as well as ventricular arrhythmias, with ventricular extrasystoles being the most frequently encountered. Holter monitoring over a period of 24–48 h is appropriate for daily arrhythmias according with current guidelines. Frequent premature ventricular contractions are defined, on ECG Holter, as more than 30 per hour during a 24-h period, or representing a premature ventricular contractions burden (percentage of total heartbeats) over 10%; on the routine ECG is considered more than 5 premature ventricular contractions per minute [64]. Complementary abnormalities identified through ECG or Holter monitoring may include atrioventricular blocks, right or left bundle branch blocks, supraventricular tachycardia, VT, and nonspecific ST-T wave changes [8].

A recent study looking at arrhythmia risk in patients with SSc and heart involvement found that sudden cardiac death occurred in a majority of cases. This revealed a striking finding, these individuals face a tenfold higher risk of sudden cardiac death compared to the general population [68].

### 5.5. Echocardiography and Other Cardiac Imaging Modality

Echocardiography remains a cornerstone in the evaluation of cardiovascular involvement in SSc, despite the availability of more advanced imaging techniques. While the underlying pathophysiology involves fibrogenesis and myocardial replacement with fibrotic tissue, studies show that only a small subset of patients with cardiac involvement exhibit a mildly reduced left ventricular ejection fraction (40–49%). A significantly reduced left ventricular ejection fraction (<40%) is rare and usually not a direct consequence of autoimmune fibromuscular degeneration alone. It also plays a critical role in identifying pericardial involvement, which is common in SSc [69]. In patients with SSc, the presence of pericardial effusion often indicates to more advanced or severe heart involvement. It may be associated with conditions like congestive heart failure, pulmonary arterial hypertension, or other forms of pulmonary vascular disease. One of the most serious and potentially life-threatening complications is when pericardial effusion occurs alongside pericarditis and cardiac tamponade: a combination that significantly raises the risk of both illness and death [69,70].

Cardiac magnetic resonance imaging has become an important supportive tool for echocardiography when a more detailed view of heart structure and function is needed. In patients with SSc, CMR has been able to detect signs of diastolic dysfunction in both ventricles, even in cases where echocardiography appears normal. One of the primary benefits of CMR is its ability to identify late gadolinium enhancement, a reliable indicator of myocardial fibrosis that might otherwise go unnoticed [71].

In a study of 344 patients with SSc, late gadolinium enhancement was found in about 25% of participants and was significantly connected to both digital ulcers and ventricular arrhythmias. Another recent study focusing on SSc patients with myocarditis highlighted the value of CMR in distinguishing between inflammatory and fibrotic lesions. Even more importantly, it was able to differentiate which of these changes were potentially reversible and which were permanent, an essential distinction for guiding treatment and assessing long-term prognosis [31].

A more recent valuable imaging tool in the evaluation of cardiac involvement in SSc is positron emission tomography. This technique offers precise analyses into both myocardial metabolism and function. In one study, patients with SSc and Raynaud’s phenomenon were found to have a significantly reduced myocardial flow reserve when examinated by Positron emission tomography. Another innovative study explored the use of positron emission tomography imaging to detect fibroblast activation with the radiotracer [(68 Ga) Ga-FAPI-04]. It found that patients with arrhythmias and elevated NT-proBNP levels showed increased radiotracer uptake, pointing to a possible connection between fibroblast activity, myocardial fibrosis, and a higher risk of arrhythmias [72,73].

### 5.6. Cardiovascular Risk Stratification Tools

At the same time, several tools have been developed to support the early identification of patients with a higher risk of cardiovascular complications. Among the most widely used are scoring systems like the Framingham Risk Score and QRISK3. These models have been adapted to autoimmune diseases to estimate long-term cardiovascular risk By estimating a person’s overall cardiovascular risk, they help guide doctors in making informed decisions about who might benefit from early preventive strategies—ultimately with the goal of reducing the chances of heart-related illness and death [31].

### 5.7. Interdisciplinary Team Need

Systemic sclerosis affects multiple organs and systems. Therefore, the authors considered that, for appropriate evaluation and interdisciplinary collaboration, the formation of a SSc team is mandatory. It may include, in addition to the rheumatologist, an internal medicine specialist, cardiologist, electrophysiologist, dermatologist, nephrologist, neurologist, the nurse and any other specialty useful to the patient. Currently, unlike in cardiology, there is no established interdisciplinary team dedicated to patients with SSc. This team would be very useful not only in monitoring the patient but especially in the early diagnosis of complications related to the severity of the disease and the iatrogenic effects of SSc therapy or comorbidities. Studies related to the evolution of these patients under the monitoring of such an SSc team are required. Accordingly, in patients with newly documented premature ventricular contractions, a baseline 12-lead ECG, long recording on 12-lead ECG, whenever possible, and an echocardiogram are recommended as first-line evaluation (class IC recommendation). Cardiac Magnetic Resonance Imaging is indicated when echocardiography does not provide sufficient information or when the presence of myocardial scarring or fibrosis is suspected. Additional investigations, such as exercise testing or electrophysiological studies, may be necessary for risk stratification and optimal treatment selection. Management is based on symptom severity, arrhythmic burden, and the presence of underlying heart disease, with the goal of reducing symptoms, preventing complications, and improving prognosis [64].

## 6. Management of Arrhythmias in Systemic Sclerosis

Taking into consideration the multiple ways that SSc can damage the heart, there is a large therapeutic arsenal that the clinician could take advantage, but it must be used wisely in order to treat each pathologic manifestation, no matter whether it is an inflammatory problem, an electrical issue or perhaps cardiac failure.

Beta-blockers can be used, should premature atrial or ventricular ectopic beats manifest in a symptomatic matter or if the amount quantified in 24 h is significant. If myocardial ischemia is also suspected, it is recommended that beta-blockers are used as a first line of treatment and this aspect should be thoroughly taken into consideration noting that coronary atherosclerosis is more frequent and more severe than in the general population. Although this medication has many uses, care should be taken as there is an important risk of exacerbating Raynaud’s phenomenon, as well as digital ulcerations. With this in mind, cardio-selective beta-blockers are definitely the preferred option and in cases of heart rates > 70/min, Ivabradine is also an option. In the event that patients develop severe bradycardia with syncope, important atrioventricular blocks (such as high-grade blocks, complete heart blocks) and other conduction disturbances, the guidelines recommend pacemaker implantation. Antiarrhythmic drugs remain a cornerstone in managing ventricular arrhythmias; however, their use is tempered by significant risks, including proarrhythmic and adverse hemodynamic effects [64].

To date, only beta-blockers have demonstrated a clear reduction in all-cause mortality. In acute scenarios such as electrical storm, combined therapy with beta-blockers and amiodarone is frequently employed to suppress recurrent arrhythmias. According to the current guidelines and the Vaughan Williams classification, amiodarone is the only antiarrhythmic agent recommended for both supraventricular and ventricular arrhythmias in patients with structural heart disease or heart failure. Class I agents, including flecainide and propafenone, are contraindicated due to proarrhythmic risk, especially in those with structural heart disease or reduced left ventricular function. Mexiletine, a Class IB agent, can be used adjunctively for VT, particularly in ischemic cardiomyopathy, though its efficacy is modest and side effects may limit its use. Sotalol, which has both beta-blocking and potassium channel-blocking properties, is utilized in VT suppression but requires careful QT interval monitoring because of the risk of torsade de pointes. Dronedarone, structurally related to amiodarone but with a more favorable side effect profile, is contraindicated in patients with NYHA class III–IV heart failure. Calcium channel blockers, such as verapamil and diltiazem, are generally not recommended for ventricular arrhythmias or heart failure with reduced ejection fraction due to their negative inotropic effects and lack of demonstrated efficacy I n preventing ventricular arrhythmias. Amiodarone decreases sinus node activity, prolongs the QT interval, and is effective in managing PVCs, VT, and VF. Despite its clinical utility, it carries a significant long-term side effect profile, including bradycardia, thyroid dysfunction, pulmonary toxicity, hepatotoxicity, and neuropathy. These risks require careful patient selection and monitoring. In SSc, where myocardial fibrosis is common and contributes to arrhythmogenesis, amiodarone is often considered the treatment of choice. However, its known pulmonary toxicity may complicate management in patients with pre-existing interstitial lung disease [64].

If malignant ventricular arrythmias appear, an implantable cardioverter defibrillator is required for sudden cardiac death prevention. According to the current guidelines on arrhythmias and sudden cardiac death, there are well-established indications for both primary and secondary prevention of sudden cardiac death. However, there are no specific studies or guideline-based indications addressing the use of implantable defibrillators or pacemakers specifically in this patient population. When arrythmias such as atrial fibrillation or atrial flutter are objectified on the surface ECG or the Holter monitoring, there is should be no holding back regarding the prescription of anticoagulants and direct oral anticoagulants should be the preferred option [31,74].

### Cardiac Complications in Systemic Sclerosis Associated with Arrhythmias

The complications associated with SSc as pericarditis and myocarditis are an important structural substrate for arrhythmias. Optimal therapy of SSc can reduce the occurrence of these cardiac complications, which are associated with a high percentage of supraventricular and ventricular arrhythmias. The first line of treatment in patients that develop symptomatic pericarditis, as in many other types of pericarditis, is considered to be NSAIDs (non-steroidal anti-inflammatory drugs). This medication proved to be effective in relieving inflammation; however, it should be used with caution in the event that patients have or develop upper gastrointestinal problems (for example gastritis), or colchicine may be instead used and even high doses of glucocorticoids. There are cases when the fluid build-up is very significant so pericardial drainage may be needed [74].

If the patient suffers from symptomatic myocarditis confirmed by CMR, it is well established in the literature that the use of immunosuppressive drugs is needed, as myocardial involvement is common and one of the leading causes of death among SSc patients. Corticosteroid therapy plays a primary role and it can be administered both orally and intravenous. Drugs such as cyclophosphamide, azathioprine, methotrexate, MM represent the pinnacle immunotherapy, used either separately or in combination with GCs. There is still the need for further studies that elaborate the topic, but as of now MMF appears to have the best safety profile and has provided the best therapeutic results, making it the most used drug out of its category [31].

Heart failure could be associated with any arrhythmia because of the high-grade level of myocardial fibrosis. Patients with SSc can develop heart failure and conventional treatment should be used as the ESCs guidelines recommend. The 4 pillars of heart failure have been very well cemented by numerous studies and include ACE inhibitors/ARBs (angiotensin II receptor blockers)/ARNI (angiotensin II receptor-neprilysin inhibitor), mineralocorticoid receptor antagonists (MRA), sodium glucose co-transporter-2 inhibitors (SGLT2i) and beta-blockers. Although this medication is safe and proved to be efficient in treating HF, there is a minor aspect that should be mentioned–SSc patients that are treated with ACE display a risk for developing SSc renal crisis. ACE are still the first line of treatment in this category, however great caution is needed when there is a high risk of developing SSc renal crisis [75].

Pulmonary hypertension could be a feared complication in SSc. All patients that develop pulmonary hypertension should be redirected to a specialized pulmonary arterial hypertension center as they require multidisciplinary coordination, having a poor prognosis long term. Thankfully there are therapeutic options that proved to be successful as well as new treatment options that provided encouraging results in multiple clinical trials, raising the bar in terms of efficiency, safety profile and tolerability Endothelin receptor antagonist Bosentan has been approved for the treatment of pulmonary arterial hypertension and has provided impressive results. It has been considered the pinnacle therapy in this category of patients and as of recently it has been certified as safe to use for patients that suffer from autoimmune diseases, more specifically connective tissue diseases like SSc. The dosage of 62.5 mg used twice a day is being up titrated to 125 mg after a month (in the mornings and evenings), however hepatic tolerance and hemoglobin levels are mandatory to be checked. This medication benefits in preventing digital ulcers making it the first line of treatment in SSc-associated pulmonary arterial hypertension. Ambrisentan is also a safe option with the advantage of being prescribed as a single pill daily (5 mg that can be increased up to 10 mg a day). To be noted that it is contraindicated in cases of pulmonary fibrosis and SSc patients do develop interstitial lung disease, requiring caution. Another class of medication used in treating SSc with pulmonary arterial hypertension is phosphodiesterase 5 inhibitors Sildenafil and Tadalafil, approved for NYHA II or III functional classes. There are no parameters to be monitored when prescribing this therapy [76,77].

Systemic sclerosis affects multiple organs and systems. Therefore, the authors considered that, for appropriate evaluation and interdisciplinary collaboration, the formation of a SSc team is mandatory. It may include, in addition to the rheumatologist, an internal medicine specialist, cardiologist, electrophysiologist, dermatologist, nephrologist, neurologist, the nurse and any other specialty useful to the patient. Currently, unlike in cardiology, there is no established interdisciplinary team dedicated to patients with SSc. This team would be very useful not only in monitoring the patient but especially in the early diagnosis of complications related to the severity of the disease and the iatrogenic effects of SSc therapy or comorbidities. Studies related to the evolution of these patients under the monitoring of such an SSc team are required. Therefore, the authors of this manuscript propose the concept of such an interdisciplinary team.

## 7. Prevention of Arrhythmias in Systemic Sclerosis

There are several essential pillars in the prevention of arrhythmias among patients with autoimmune rheumatic diseases, particularly SSc. The foremost and perhaps most critical is cardiac screening and risk stratification. If implemented consistently, this approach could potentially alter the disease trajectory and prevent abrupt progression. The main diagnostic methods for identifying potential cardiac involvement in SSc include routine cardiology consultations, standard ECG, and echocardiographic evaluation of both structural and functional cardiac abnormalities. These tools facilitate the early detection and management of arrhythmias associated with SSc.

A prompt and accurate management of the diverse manifestations of cardiac involvement forms the second pillar of prevention. Initiating immunosuppressive therapy to control both inflammation and fibrosis—the pathological hallmarks of SSc—has been shown to reduce the risk of arrhythmia development. Moreover, antifibrotic agents used primarily for pulmonary fibrosis have demonstrated indirect benefits in attenuating myocardial fibrosis, further supporting their role in cardiac protection.

Given that dysfunction in one organ system often leads to secondary involvement of others, effective management of comorbidities is crucial, especially in this patient category. Additionally, careful selection of pharmacological therapies can significantly influence disease outcomes. For instance, the use of beta-blockers must be judicious, as they may exacerbate Raynaud’s phenomenon which is a frequent complication in SSc.

Lastly, but equally important, is patient education. Informing patients about the potential cardiac symptoms and complications associated with SSc can promote early referral to cardiology specialists and facilitate timely therapeutic intervention and disease monitoring.

## 8. Future Perspectives

Advancements in diagnostic modalities, such as cardiac magnetic resonance imaging and extended ambulatory heart monitoring, hold promise for the earlier detection of myocardial involvement and arrhythmias in SSc. These innovations enable identification of subclinical cardiac fibrosis and transient rhythm disturbances, facilitating timely intervention before overt clinical manifestations occur. On the therapeutic front, ongoing research is focused on refining immunomodulatory and antifibrotic strategies aimed at halting or reversing myocardial damage. Moreover, the integration of novel arrhythmia management techniques, including catheter ablation and implantable cardiac devices, offers improved outcomes for patients with refractory arrhythmias.

Future care models emphasize a multidisciplinary approach involving rheumatologists, cardiologists, electrophysiologists, pulmonologists, dermatologists, gastroenterologists, nephrologists, internal medicine and rehabilitation specialists, alongside nursing professionals, to deliver comprehensive and personalized management. Such collaborative frameworks are essential to optimize early diagnosis, tailor treatments, and ultimately improve prognosis and quality of life in patients with SSc-related cardiac complications.

## 9. Conclusions

The “heart team” paradigm, a well-recognized model within cardiology, exemplifies the critical role of multidisciplinary collaboration in managing complex cardiovascular conditions. In the context of SSc, the integration of a multidisciplinary team encompassing rheumatologists, cardiologists, electrophysiologists, pulmonologists, internal medicine and other relevant specialists facilitates comprehensive evaluation and management. Such interdisciplinary cooperation enables earlier detection of cardiac involvement, precise quantification of disease severity, and the implementation of individualized therapeutic strategies. These factors collectively contribute to improved risk stratification and may substantially modify the clinical trajectory and prognosis of patients with SSc-related cardiac complications. Consequently, the adoption of coordinated, team-based approaches represents a pivotal advancement in optimizing patient outcomes in this multifaceted disease.

## Figures and Tables

**Table 1:** Potential Cardiovascular Risk Factors and Arrhythmias.

Authors/Year	Risk/Feature	Mechanism	Clinical Effects
Khaled M. Othman et al. (2010) [17]	Autonomic dysfunction	Presence of imbalance between the sympathetic/parasympathetic system.	The risk of arrhythmias and sudden cardiac death is increased.
Nadel. A et al. (2024) [31]	Myocardial fibrosis	Chronic inflammation and ischemia caused by the collagen deposition.	Restrictive cardiomyopathy; Heart failure.
Nadel. A et al. (2024) [31]	Medication effects	Cardiotoxic or arrhythmogenic side effects.	Increased cardiovascular risk
Xintao Cen et al. (2020) [32] Francesca C. et al. (2015) [33]	Microvascular and Macrovascular ischemia	Inflammatory process; Endothelial dysfunction; Coronary vessel involvement.	Death due to cardiac causes (26% patients with SSc)-heart failure and arrythmia;
Nabil EL-Sherif et al. (2011) [34]	Electrolyte imbalances	Chronic inflammation; Renal dysfunction; Gastrointestinal involvement; Medication effects.	Torsade de pointes; Ventricular arrhythmias-asystole/cardiac arrest; Refractory arrhythmias; Heart failure.

**Table 2:** Effects of immunosuppressive medication in SSc and on cardiovascular system. AZA, Azathioprine; CYC, Cyclophosphamide; ILD, Interstitial Lung Disease; MMF, Mycophenolate mofetil; MTX, Methotrexate.

Authors/Year	Therapeutic Class	Effects in Systemic Sclerosis	Cardiovascular Effects	
			Benefits	Risks
IMMUNOSUPRESSANTS				
Toshinnori Takada et al. (2024) [35] Misbah Baqir et al. (2017) [36] Moradi Z. et al. (2025) [37]	Mycophenolate mofetil	First line for SSc-ILD Improves cutaneous fibrosis	The evidence regarding cardiovascular benefits remains limited.	Rare arrhythmias reported (supraventricular tachycardia, atrial and ventricular ectopic, bundle branch block).
Tracy M. Frech et al. (2013) [38] Sareena Shah et al. (2022) [39]	Methotrexate	Early diffuse cutaneous SSc (alternative, not first-line). Modestly reduce skin thickening.	Although some studies suggest potential effects, the evidence is not sufficient to confirm cardiovascular benefits.	Rare cases with ventricular arrhythmias, right bundle branch block, and other idiosyncratic rhythm disturbances, heart failure.
Ege Sinan T. et al. (2025) [40]	Azathioprine	Alternative therapy (when MMF/CYC are contraindicated). Positive results in stabilizing/improving ILD as maintenance after Cyclophosphamide Improvement in skin fibrosis after prior Cyclophosphamide.	Evidence for cardiovascular benefit remains inconclusive.	Possibly myocarditis/toxic myocardial injury.
Baron F. et al. (2018) [41] Agarwal N. et al. (2013) [42] Nadel A. et al. (2024) [31]	Cyclophosphamide	Historically first-line for SSc-ILD; effective in severe diffuse cutaneous SSc. Improves forced vital capacity in SSc-ILD (short-term benefit). Improves skin fibrosis-effect similar to MMF.	Can reduce inflammation in SSc myocarditis (indirect effect).	High doses lead to atrial fibrillation, supraventricular tachycardia, premature ventricular contractions, ventricular tachycardia, high-grade atrioventricular bloc, cardiomyopathy and heart failure.

**Table 3:** Effects of corticosteroids in systemic sclerosis and on cardiovascular system. ILD, Interstitial Lung Disease.

Authors/Year	Effects in Systemic Sclerosis	Cardiovascular Effects	
		Benefits	Risks
CORTICOSTEROIDS			
Papadimitriou T. et al. (2022) [43] Stroeder Jasmine et al. (2015) [44] Alessandra Vacca et al. (2013) [8] Muhammed Recai Akdoğan et al. (2023) [45]	Overlap syndromes (e.g., SSc-myositis)—not recommended for long-term Reduce early inflammatory skin edema; limited long-term effect on fibrosis Used in SSc-ILD, often combined with immunosuppressants	Can reduce myocardial/pericardial inflammation	Arrhythmias: bradycardia (high-dose pulse > 250 mg/day), ventricular arrhythmias (premature ventricular contractions, no sustained ventricular tachycardia, ventricular tachycardia), atrial arrhythmias (atrial fibrillation, atrial flutter); electrolyte disturbances (hypokalemia, hypocalcemia); can worsen pre-existing myocardial fibrosis SSc renal crisis: doses > 15 mg/day for ≥6 months increase SSc renal crisis risk to ~36% in diffuse cutaneous SSc.

**Table 4:** Therapeutic implications of vasodilators in systemic sclerosis and cardiovascular manifestations.

Authors/Year	Therapeutic Class	Effects in Systemic Sclerosis	Cardiovascular Effects	
			Benefits	Risks
VASODILATOR THERAPY				
Alexis F. Guédon et al. (2024) [46] F. Guideri et al. (2006) [47] Sevdalina Lambova et al. (2014) [53]	Calcium channel blockers (e.g., Nifedipine, Amlodipine, and Nicardipine)	First-line in Raynaud’s phenomenon and digital ulcers;	Improves myocardial perfusion in microvascular coronary involvement and cardiac function. Dihydropyridine calcium channel blockers-minimal negative inotropic effects.	Reflex tachycardia, hypotension, rare arrhythmogenic effects
VK Bournia et al. (2018) [48] AF Guedon et al. (2024) [46]	PDE-5 inhibitors (e.g., Sildenafil and Tadalafil)	First-line for pulmonary hypertension Considered second-line for digital ulcers	Improve diastolic function and left ventricular ejection fraction Potential myocardial protective effect.	Tachycardia, hypotension, palpitations Arrhythmogenic potential minimal-no conclusive evidence confirms the arrhythmogenic potential Contraindicated in unstable angina or recent myocardial infarction
VK Bournia et al. (2018) [48]	Prostacyclin analogues (e.g., Iloprost and Esoprostenol)	Treat severe Raynaud’s phenomenon, digital ulcers and pulmonary hypertension. Improve microvascular perfusion.	Prostaglandins are involved in cardiovascular physiology, yet evidence of clear clinical benefits remains insufficient	Can prolong ventricular refractory period and QTc; myocardial “steal” phenomenon → ischemia. Contraindicated in severe coronary artery disease, thrombotic events, congestive heart failure, severe arrhythmias; hypotension.
LM. Lammi et al. (2025) [49] Cacciapaglia F. et al. (2025) [50] Matucci-Cerinic et al. (2011) [51] Silva I. et al. (2015) [52]	Endothelin receptor antagonists (e.g., Bosentan and Ambrisentan)	Reduce new digital ulcers Improve microvascular function	Potential benefit in myocardial perfusion	Hypotension Arrhythmogenic potential not clearly demonstrated

**Table 5:** Cardiovascular and systemic sclerosis effects of ACE inhibitors therapy. ACE, Angiotensin-converting enzyme.

Authors/Year	Effects in Systemic Sclerosis	Cardiovascular Effects	
		Benefits	Risks
ACE INHIBITORS (e.g., Ramipril, Fosinopril, and Trandolapril)			
Sevdalina Lambova et al. (2014) [53] Anji Xiong et al. (2022) [54]	First-line therapy in SSc renal crisis	Renal protection-improves cardiovascular effects (indirect benefit)	Hypotension Hyperkalemia

**Table 6:** The role and the effects of antifibrotic agents in systemic sclerosis and cardiovascular system. ILD, Interstitial Lung Disease.

Authors/Year	Effects in Systemic Sclerosis	Cardiovascular Effects	
		Benefits	Risks
ANTIFIBROTIC AGENTS (e.g., Nintedanib)			
Yannick Allanore et al. (2025) [55] Derrick Herman et al. (2024) [56] James R. Siebold et al. (2020) [57] Assassi S. et al. (2022) [58] Ninagawa K. et al. (2023) [59]	Approved for SSc-ILD-slows progression of lung function. Can be used alone/in combination with immunosuppressants. Limited evidence on skin fibrosis.	Potential cardioprotective effect: reduced myocardial inflammation/fibrosis, improved right ventricular function.	Minimal cardiovascular risk reported in trial.

**Table 7:** Biologic therapy: implications in systemic sclerosis and cardiac effects. AZA, Azathioprine; CYC, Cyclophosphamide; ILD, Interstitial Lung Disease; MMF, Mycophenolate mofetil.

Authors/Year	Therapeutic Class	Effects in Systemic Sclerosis	Cardiovascular Effects	
			Benefits	Risks
BIOLOGIC THERAPY				
Santis M. et al. (2025) [60] Adjailia EB et al. (2025) [61]	Rituximab (anti-CD20)	Therapy in skin fibrosis, SSc-ILD, SSc-myocarditis Especially in cases refractory to CYC or MMF	In patients with primary cardiac involvement: improved cardiac function, reduction in myocardial inflammation and fibrosis; potential adjunct in SSc-related myocarditis	Arrhythmogenic risk not fully documented; larger studies needed
Kuster S. et al. (2022) [62] Zhong-Chao Fu et al. (2025) [63]	Tocilizumab (IL-6 receptor blocker)	Slowing progression of cutaneous and pulmonary involvement Adjunct therapy in non-responsive or severe cases	Stabilization of cardiac symptoms, improvement in myocardial involvement when combined with immunosuppressants (e.g., CYC)	Rare reports of atrial flutter, heart failure, and sudden death; Arrhythmias-not reported

## Data Availability

No new data were created or analyzed in this study.

## Abbreviations

The following abbreviations are used in this manuscript:

SSc	Systemic sclerosis
SSc-ILD	SSc-Associated Interstitial Lung Disease
AF	Atrial Fibrillation
Ang I	Angiotensin I
Ang II	Angiotensin II
ET-1	Endothelin-1
CMR	Cardiovascular Magnetic Resonance Imaging
ANS	Autonomic Nerve System
sPAP	Pulmonary Artery Pressure
HRT	Heart Rate Turbulence
RAAS	Renin–Angiotensin–Aldosterone System
ACE	Angiotensin-Converting Enzyme
ACE 2	Angiotensin-Converting Enzyme 2
EAT	Epicardial Adipose Tissue
GCs	Glucocorticoids
MMF	Mycophenolate mofetil
MTX	Methotrexate
AZA	Azathioprine
CYC	Cyclophosphamide
mRSS	Modified Rodnan Skin Score
HF	Heart Failure
AT2R	Angiotensin II Type 2 Receptor
RVSP	Systolic Pulmonary Artery Pressure
dcSSc	Diffuse Cutaneous SSc
